# Inhibition of PI3K Prevents the Proliferation and Differentiation of Human Lung Fibroblasts into Myofibroblasts: The Role of Class I P110 Isoforms

**DOI:** 10.1371/journal.pone.0024663

**Published:** 2011-10-03

**Authors:** Enrico Conte, Mary Fruciano, Evelina Fagone, Elisa Gili, Filippo Caraci, Maria Iemmolo, Nunzio Crimi, Carlo Vancheri

**Affiliations:** Department of Clinical and Molecular Biomedicine, University of Catania, Catania, Italy; National Jewish Health, United States of America

## Abstract

Idiopathic pulmonary fibrosis (IPF) is a progressive fibroproliferative disease characterized by an accumulation of fibroblasts and myofibroblasts in the alveolar wall. Even though the pathogenesis of this fatal disorder remains unclear, transforming growth factor-β (TGF-β)-induced differentiation and proliferation of myofibroblasts is recognized as a primary event. The molecular pathways involved in TGF-β signalling are generally Smad-dependent yet Smad-independent pathways, including phosphatidylinositol-3-kinase/protein kinase B (PI3K/Akt), have been recently proposed. In this research we established *ex-vivo* cultures of human lung fibroblasts and we investigated the role of the PI3K/Akt pathway in two critical stages of the fibrotic process induced by TGF-β: fibroblast proliferation and differentiation into myofibroblasts. Here we show that the pan-inhibitor of PI3Ks LY294002 is able to abrogate the TGF-β-induced increase in cell proliferation, in α- smooth muscle actin expression and in collagen production besides inhibiting Akt phosphorylation, thus demonstrating the centrality of the PI3K/Akt pathway in lung fibroblast proliferation and differentiation. Moreover, for the first time we show that PI3K p110δ and p110γ are functionally expressed in human lung fibroblasts, in addition to the ubiquitously expressed p110α and β. Finally, results obtained with both selective inhibitors and gene knocking-down experiments demonstrate a major role of p110γ and p110α in both TGF-β-induced fibroblast proliferation and differentiation. This finding suggests that specific PI3K isoforms can be pharmacological targets in IPF.

## Introduction

Idiopathic pulmonary fibrosis (IPF) is a interstitial lung disease characterized by aberrant matrix deposition and destruction of the normal lung architecture [1]. Survival of IPF patients is poor, with a 5-year survival rate of only 20% [2]. IPF has historically been treated with corticosteroids and/or cytotoxic agents such as prednisone without any evidence-based benefit. Given the inefficacy of conventional therapies, novel strategies are required for the management of IPF as well as a better understanding of the molecular mechanisms underlying the pathogenesis and progression of this disease. A determinant role in IPF is played by myofibroblasts, as these cells, characterized by α–smooth muscle actin (α-SMA) fibres, have a contractile phenotype and abundantly synthesize collagen and ECM proteins [3]. Myofibroblasts may be derived by activation/proliferation of resident lung fibroblasts, epithelial-mesenchymal differentiation, or recruitment of circulating fibroblastic stem cells (fibrocytes). Transforming growth factor-β1 (TGF-β1) is known to induce the differentiation of human lung fibroblasts into myofibroblasts [4], [5]. However, the molecular pathways involved in TGF-β-induced myofibroblast transformation have only been partially identified and Smad-dependent as well as independent pathways, including PI3K, have been proposed [6]–[8]. PI3K is a signal transduction enzyme that catalyzes the phosphorylation of phosphatidylinositol (4,5)-biphosphate to form phosphatidylinositol (3,4,5)-triphosphate in response to the activation of receptor tyrosine kinases, G protein coupled receptors/cytokine receptors and activated Ras. PI3K signalling has been implicated in the control of a wide range of cellular activities such as proliferation, survival, adhesion, differentiation, cytoskeletal organization, etc. [9], [10]. PI3Ks have been divided into three classes according to their structure and lipid substrate specificity. The most extensively investigated are the class I PI3Ks that act on PI-(4,5)-bisphosphate (PIP2) to produce PI-(3,4,5)-triphosphate (PIP3). Prototypical class I PI3K is a dimeric enzyme, consisting of catalytic and regulatory subunits. The catalytic subunit occurs in four isoforms, designated as p110α, p110β, p110γ and p110δ, which are sub-grouped into Class IA (p110α, p110β and p110δ) and IB (p110γ). These isoforms have been demonstrated to have both overlapping and unique roles in physiology and disease states. To date, both genetic manipulation and pharmacological inhibitors have been utilized to understand the roles of individual PI3K isoforms and distinct kinase functions as well as kinase-independent functions have been revealed.

In this research we investigated the role of the PI3K pathway in the TGF-β-induced proliferation of *ex-vivo* human lung fibroblasts and their differentiation into myofibroblasts. Moreover, by using selective inhibitors of class I PI3K p110 isoforms as well as specific gene suppression by small interfering RNA (siRNA) we further identified the contribution of individual p110 isoforms to these processes.

## Materials and Methods

### Ethics Statement

The Italian and institutional policies of humane care have been abided by conscientiously and our study was approved by the Azienda Ospedaliera ‘Garibaldi, S.Luigi-Currò, Ascoli-Tomaselli’ ethical committee. Written informed consent was obtained from all participants involved in the study.

### PI3K inhibitors

LY294002 was from Sigma, AS-252424 and TGX-221 were from Enzo Life Sciences AG (Lausen, Switzerland), IC87114 from BioVision (Mountain View, CA, USA), YM-024 was kindly provided by Prof. Shaun P. Jackson, Australian Centre for Blood Diseases, Monash University, Melbourne, Australia). TGF-β was from Chemicon. All others reagents were from Sigma.

### Cell culture and treatments

Lung fibroblast cells were derived from histologically normal areas of surgical lung specimens from patients undergoing resective surgery for benign or malignant tumors. Primary lines were established by using an outgrowth from explants according to the method by Jordana and coworkers [11] as previously described [12]. In all the experiments, cell lines were used at a passage earlier than the height. Prior to treatment, cells were incubated for 24 hrs in serum-free RPMI medium, then left resting or treated with various PI3K inhibitors one hour before subsequent TGF-β stimulation (10 ng/ml, Chemicon) in the absence or presence of PI3K inhibitors. Afterward, cells were incubated for 24 or 48 hrs in serum-free medium. All of the phenotypic and functional parameters were then evaluated. The cytotoxicity of all substances was evaluated by the LDH cytotoxicity detection kit (Roche). Transfections with commercially available siRNA specific for PI3K p110 isoforms α and γ as well as with a negative control (Qiagen, Flexi Tube Gene Solution SI00605843, SI02665369 and AllStars Negative Control, respectively) were performed in serum free for 24 hours by using siPORT™ Amine Transfection Agent (Ambion, Inc.) following manufacturer's protocol. Afterward transfection medium was replaced and cells were stimulated or not with TGF-β for 24 hours in 2% FBS medium.

### Cell proliferation

Cell numbers were determined by counting cells on a hemocytometer (Burker chamber) after trypan blue staining. A mean of four fields was used to calculate the average number of cells. Cell proliferation was also evaluated by using the cell proliferation WST-1 kit (Roche, Basel, Switzerland). Briefly, after the specified treatment, cells were exposed to WST-1 for 1 hour at 37°C. The formation of WST-1 formazan was spectrophotometrically monitored using a reference wavelength of 480 nm.

### Collagen production

TGF-β–stimulated fibroblasts or resting cells, cultured in the absence or presence of inhibitors, were scraped into a lysis buffer..Total soluble collagen was measured by the Sircol Soluble Collagen Assay (Biocolor, Newtownabbey, United Kingdom). The collagen-dye complex was precipitated by centrifuging at 10,000×g for 10 minutes. The precipitated complex was resuspended in 1 ml alkali reagent. The solution obtained was finally placed in a 96-well flat-bottomed plate and evaluated in a plate reader (absorbance, 540 nm).

### Flow-cytometry analysis of α-SMA expression

The cells were washed, fixed with paraformaldehyde (PFA) 2%, and permeabilized with Triton 1× (Sigma-Aldrich). The cells were then incubated for 60 minutes with α-SMA-ab (1∶200; DAKO, Glostrup, Denmark), anti-hTGF-b1 (1∶200; R&D Systems, Minneapolis, Minn). Subsequently, the cells were washed once with PBS/BSA 1% and incubated with Goat F(ab′) 2 Fragment Anti-Mouse IgG (H1L)-FITC (Beckman Coulter, Milano, Italy). Samples were analyzed by using a Coulter Epics Elite ESP flow cytometer (Coulter Corp, Miami, Fla).

### RNA extraction and RT-PCR

Total RNA from cells was extracted by using TRIZOL reagent (Invitrogen Inc., Paisley, UK), quantified by specrophotometric analysis with a BIO-photometer (Eppendorf, Germany) and treated with DNAse (Invitrogen, UK). The generation of cDNA from RNA (2 µg) was performed with Superscript™ II Reverse Transcriptase (RT, Invitrogen, UK) and random hexamer primers (Invitrogen, UK), according to the manufacturer's instructions. Quantitative real-time PCR of cDNAs was performed using the IQ SYBR Green Supermix (QIAGEN, Germany) in conjunction with commercially available GAPDH, PI3K p110γ (QIAGEN) and PI3K p110δ assays (Applied Biosystems), according to the manufacturer's instructions. PCR amplicons were also run in 2% agarose gel and visualized by sybr-safe DNA stain (Invitrogen).

### Western blot analysis

Resting, treated and/or transfected cells were lysed in a solution of 10 mM EDTA, 1% Triton x-100, 1 mM PMSF (phenylmethylsulfonylfluoride) and 15 µl/ml Protease Inhibitor Cocktail (all from Sigma-Aldrich Corp.). Protein concentrations were determined by the Bradford method. Samples were then diluted in sample buffer and boiled for 5 min. Electrophoresis was performed on a 12% SDS-PAGE gel (40 mA/h) using 60 µg of proteins/lane. After electrophoresis, the proteins were transferred onto a nitrocellulose membrane (Hybond ECL, Amersham Biosciences Europe GmbH, Milan, Italy) for 2 hrs at room temperature with a transblot semidry transfer cell. After BSA blocking, the membranes were incubated overnight at 4°C with: monoclonal mouse anti- hα-SMA Ab (1∶500, Dako Cytomation, Denmark), or rabbit anti hp(Ser^473^)-Akt (1∶200; Cell Signaling Technology, Beverly, MA), monoclonal rabbit anti hPI3K p110α (1∶500, Cell Signalling), monoclonal rabbit anti h-PI3K p110β (1∶500, Abcam), monoclonal mouse anti hPI3K p110γ (1∶500, Abcam), monoclonal rabbit anti hPI3K p110δ (1∶500, Abcam), monoclonal mouse anti hGAPDH (1∶ 200, Chemicon) . Membranes were then thoroughly washed and incubated with horseradish peroxidase-conjugated anti-mouse or anti-rabbit secondary antibodies (1∶5,000; Santa Cruz Biotechnology, Inc., California, USA) . Specific bands were visualized using the Quantum Dot detection system (Invitrogen).

### Statistical analysis

Statistical significance across treatment groups was determined by using the one-way ANOVA with Tukey's multiple-comparison with Statgraphic Centurion XV software (StatPoint Technologies Inc., ADALTA, Italy). A *P* value<0.05, which indicates a statistically significant difference, is designated with a single asterisk.

## Results

### Role of PI3K/Akt pathway in TGF-β-induced proliferation and differentiation into myofibroblast

**Figure 1:**
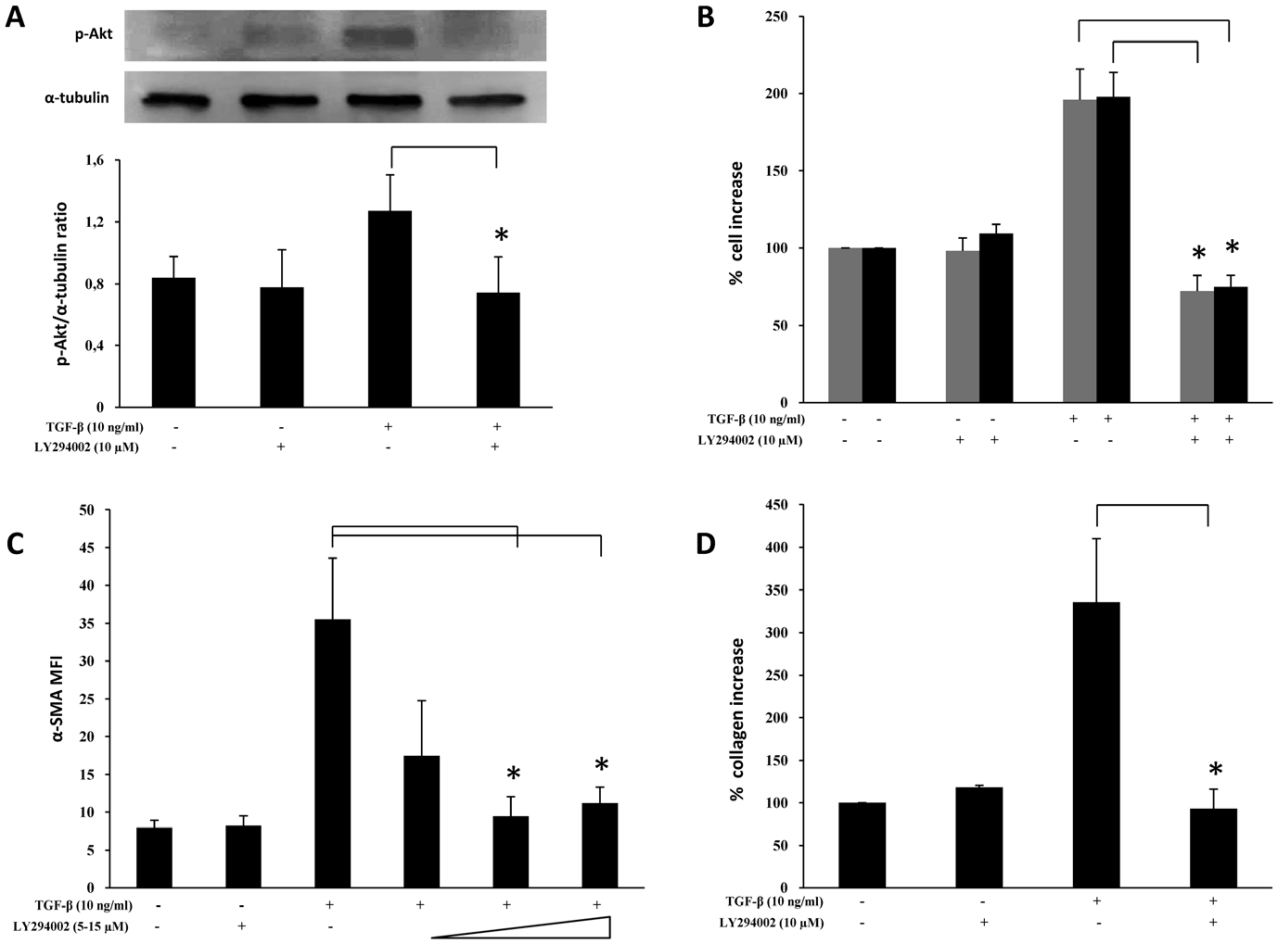
Effects of PI3Ks pan-inhibitor LY294002 on TGF-β-induced proliferation of human lung fibroblasts and their differentiation into myofibroblasts. *Ex vivo* human lung fibroblasts were cultured in vitro and all cell lines (from five different donors) were used at a passage earlier than eight. Prior to treatment, cells were incubated for 24 hrs in serum-free RPMI medium, then left resting or treated with LY294002, at indicated concentrations, one hour before subsequent TGF-β stimulation (10 ng/ml) in the absence or presence of LY294002. Afterward, cells were incubated for 48 hrs in serum-free medium and then harvested. A) Representative western blot analysis of phosphorylated (Ser^473^) Akt and pertinent graph with the pAKT/α-tubulin ratios of five separate experiments. B) Results of cell counting by optical microscopy (grey columns) and WST-1 cell proliferation assay (black columns) were represented as % increase over control (untreated cells, assumed as 100%). C) Flow-cytometry analysis of α-SMA expression: mean values (± sd) of Mean Fluorescence Intensity (MFI) are indicated. Plots of one representative experiment are shown in FigureS1. D) Total collagen levels, measured by the Sircol Soluble Collagen Assay, were reported as % increase over control. Statistical significance across treatment groups was determined using the one-way ANOVA. A *P* value <0.05, which indicates a statistically significant difference among relevant groups, is designated with an asterisk.

TGF-β is a potent paracrine mediator of myofibroblast differentiation and contributes to the development of pulmonary fibrosis following the expansion of lung myofibroblasts. Therefore, when e*x-vivo* human lung fibroblasts were treated with TGF-β (10 ng/ml) in serum free conditions for 48 hours, as expected, cells exhibited a faster proliferation rate and differentiated toward a myofibroblast phenotype characterized by α-SMA expression and collagen production, as shown in Figure 1. Moreover, as a consequence of TGF-β treatment, levels of pAKT increased (Figure 1A). Since all such effects were abrogated by co-treatment with LY294002, the broad spectrum inhibitor of the PI3K signalling pathway (Figure 1A–D), it is evident that PI3K/AKT activation induced by TGF-β plays a central role in both the proliferation of human lung fibroblasts as well as their differentiation into myofibroblasts.

### Expression of class I PI3Ks in human ex-vivo lung fibroblasts

**Figure 2:**
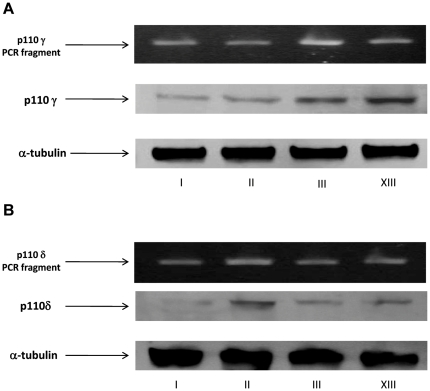
Expression of PI3Kclass IA p110δ and class IB p110γ in human ex-vivo lung fibroblasts. A) RT-PCR products of PI3K p110δ run in 2% agarose gel (upper line) and western blot analysis of proteins from four different fibroblast cell lines (medium line). The protein loading control was α-tubulin (bottom line). A commercially available RT-PCR kit and a monoclonal rabbit anti human PI3K p110δ (1∶500) were used as detailed in the Mat and Met section. B) RT-PCR products of PI3K p110γ run in 2% agarose gel and western blot analysis of proteins from the same fibroblast cell lines indicated in panel A. A commercially available RT-PCR kit and a monoclonal mouse anti human PI3Kp110γ (1∶500) were used.

LY294002 is a pan inhibitor of all four class I PI3Ks, yet it has been generally recognized that only p110α and p110β are ubiquitously expressed whereas p110δ and p110γ are restricted to haematopoietic cell lineages. Therefore, we also wanted to ascertain the expression of p110δ and p110γ in human lung fibroblasts. We performed RT-PCR as well as western blot analysis. As shown in Figure 2, the results demonstrate that both p110δ and p110γ are expressed at mRNA and protein levels in human lung fibroblasts.

### Effects of pharmacological inhibition of specific class I PI3K p110 isoforms

Since in several models, including lung disease, a unique biological activity has been shown for different class I PI3Kp110 isoforms, we wondered if they could also play specific roles in lung fibroblast proliferation and differentiation into the myofibroblasts induced by TGF-β. Specific inhibitors of class IA PI3K isoforms (YM-024, TGX-221 and IC87114 for p110α, p110β and p110δ, respectively) and class IB (AS-252424 for p110γ) were used to dissect the specific role of each isoform. Concentrations around each IC_50_ in a range of non-overlapping effects, were utilized according to literature data [13]–[16]. All the drugs were checked for cytotoxicity by an LDH test and no significant toxicity was observed for any inhibitor in the used range (data not shown).

**Figure 3:**
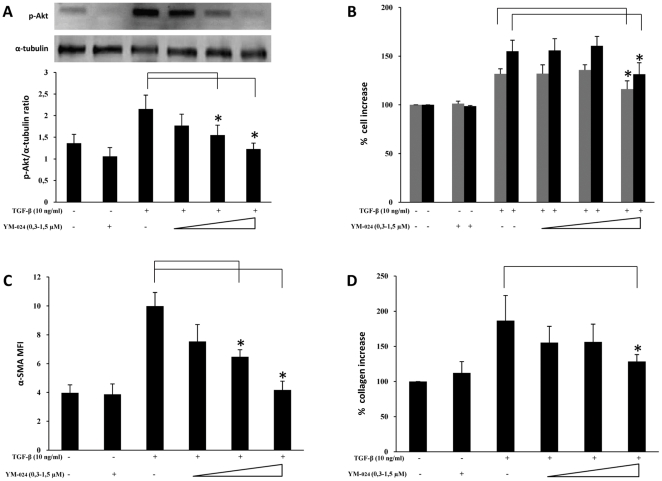
Effects of selective PI3K p110α inhibition. *Ex vivo* human lung fibroblasts (from three different donors) were cultured and treated with YM-024 following the protocol indicated in Figure 1. A) Representative western blot analysis of phosphorylated (Ser^473^) Akt and pertinent graph with pAKT/α-tubulin ratios of three separate experiments. B) Results of cell counting by optical microscopy (grey columns) and WST-1 cell proliferation assay (black columns). C) Flow-cytometry analysis of α-SMA expression: MFI mean values (± sd) of three separate experiments are indicated. Plots of one representative experiment are shown in FigureS2. D) Total collagen levels were reported as % increase over control. Statistical significance across treatment groups was determined using the one-way ANOVA. A *P* value <0.05, which indicates a statistically significant difference among relevant groups, is designated with an asterisk.

**Figure 4:**
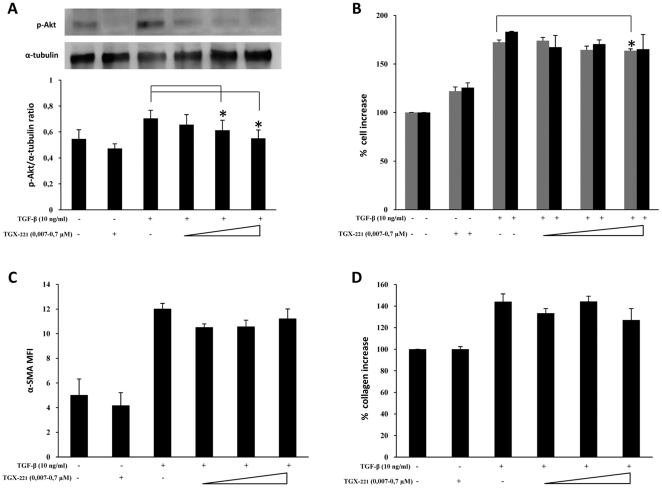
Effects of selective PI3K p110β inhibition. *Ex vivo* human lung fibroblasts (from three different donors) were cultured, and treated with TGX-221 following the protocol indicated in Figure 1. A) Representative western blot analysis of phosphorylated (Ser^473^) Akt and pertinent graph with pAKT/α-tubulin ratios of three separate experiments. B) Results of cell counting by optical microscopy (grey columns) and WST-1 cell proliferation assay (black columns) were represented as % increase over control. C) Flow-cytometry analysis of α-SMA expression: MFI mean values (± sd) of three separate experiments are indicated. Plots of one representative experiment are shown in FigureS3. D) Total collagen levels, were reported as % increase over control. Statistical significance across treatment groups was determined using the one-way ANOVA. A *P* value <0.05, which indicates a statistically significant difference among relevant groups, is designated with an asterisk.

**Figure 5:**
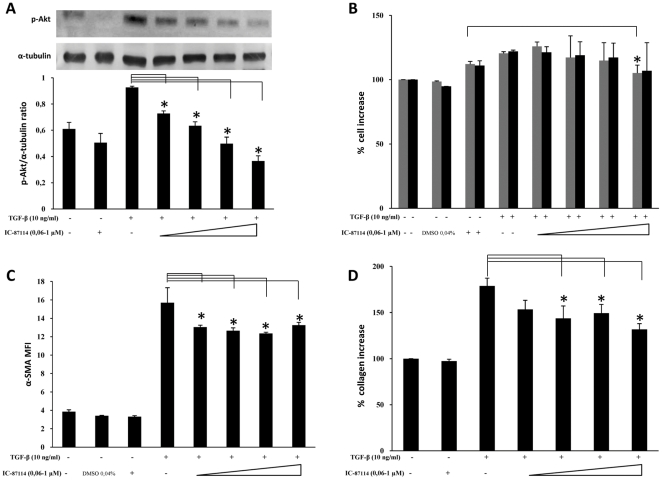
Effects of selective PI3K p110δ inhibition. *Ex vivo* human lung fibroblasts (from three different donors) were cultured, and treated with IC-87114 following the protocol indicated in Figure 1. A) Representative western blot analysis of phosphorylated (Ser^473^) Akt and pertinent graphic with pAKT/α-tubulin ratios of three separate experiments. B) Results of cell counting by optical microscopy (grey columns) and WST-1 cell proliferation assay (black columns) were represented as % increase over control. C) Flow-cytometry analysis of α-SMA expression: MFI mean values (± sd) of three separate experiments are indicated. Plots of one representative experiment are shown in FigureS4. D) Total collagen levels were reported as % increase over control. Statistical significance across treatment groups was determined using the one-way ANOVA. A *P* value <0.05, which indicates a statistically significant difference among relevant groups, is designated with an asterisk.

**Figure 6:**
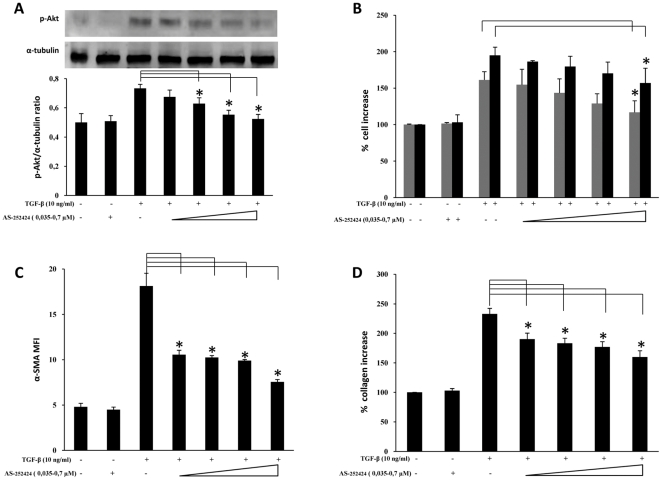
Effects of selective PI3K p110γ inhibition. *Ex vivo* human lung fibroblasts (from three different donors) were cultured, and treated with AS-2524224 following the protocol indicated in Figure 1. A) Representative western blot analysis of phosphorylated (Ser^473^) Akt and pertinent graph with pAKT/α-tubulin ratios of three separate experiments. B) Results of cell counting by optical microscopy (grey columns) and WST-1 cell proliferation assay (black columns) were represented as % increase over control. C) Flow-cytometry analysis of α-SMA expression: MFI mean values (± sd) of three separate experiments are indicated. Plots of one representative experiment are shown in FigureS5. D) Total collagen levels, were reported as % increase over control. Statistical significance across treatment groups was determined using the one-way ANOVA. A *P* value <0.05, which indicates a statistically significant difference among relevant groups, is designated with an asterisk.

As shown in Figure 3, blocking p110α activity by treatment with YM-024 was able to dose-dependently suppress the TGF-β–induced increase in both pAkt and α-SMA expression levels (panel A and C, respectively). Moreover, significant inhibition of cell proliferation and collagen production was achieved at high doses (panel B and D, respectively). By contrast, treatment with the specific p110β inhibitor TGX-221, on one hand was able to significantly inhibit TGF-β–induced Akt activation (Figure 4A) and B, respectively) yet on the other hand it was only able in part to reduce cell proliferation rates, at high doses, and it did not produce any significant variation of either α-SMA expression or collagen deposition (Figure 4, panel C and D, respectively). Differently, suppression of p110δ activity by IC87114 that dose-dependently prevented Akt phosphorylation was also able to inhibit α-SMA expression and collagen deposition, yet without any dose-dependence, besides marginally affecting the proliferation rate, as shown in Figure 5. Finally, the effects of class IB p110γ suppression by AS-252424 are demonstrated in Figure 6 which shows a blockade of Akt activation paralleled by a dose-dependent decrease in cell proliferation rate as well as α-SMA expression and collagen deposition. The extent of these effects, less so than in the case of LY294002 but comparable to that observed in the case of p110α inhibition, therefore suggests a complementary role of these isoforms. Interestingly, by using AS-252424 at a concentration ≥5 µmolar, probably affecting also p110α, the TGF-β-induced effects were completely suppressed (data not shown).

### Effects of selective gene suppression of p110 isoforms α and γ

In order to confirm the results obtained by pharmacological inhibition we performed a specific gene suppression by transfecting cells with small interfering RNAs (siRNAs) specifically targeting p110α and p110γ RNAs as well as with a negative control with no homology to any known mammalian gene.

**Figure 7:**
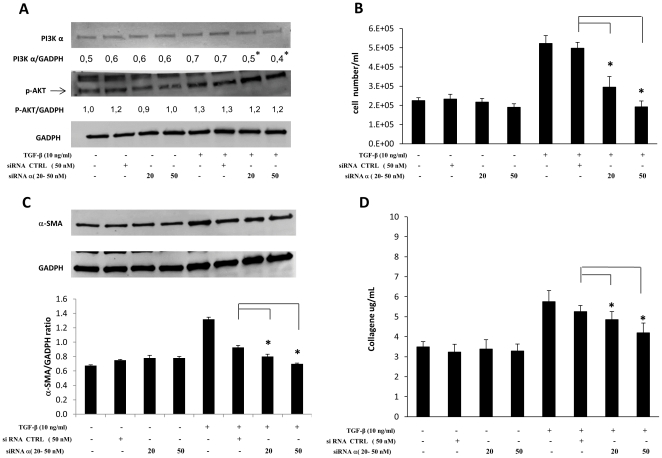
Effects of specific PI3K p110α gene suppression by siRNA. *Ex vivo* human lung fibroblasts (from three different donors) were kept resting or transfected with small interfering RNAs (siRNAs) specifically targeting p110α (at the concentrations of 20 and 50 nM) or with negative control (50 nM), for 24 hrs in serum-free RPMI medium, afterward they were kept resting or stimulated with TGF-β (10 ng/ml) for 24 hrs in 2% FBS medium. A) Representative western blot analysis of p110α, phosphorylated (Ser^473^) Akt and GAPDH expression. with means of p110α-/GAPDH and pAKT/α-GAPDH ratios calculated in three separate experiments B) Results of cell counting by optical microscopy. Medians and standard deviations of at least three separate counts in triplicate wells for three separate experiments are reported. C) Representative Western blot analysis of α-SMA expression. and pertinent graph with means of α-SMA/GAPDH ratios calculated in three separate experiments. D) Total soluble collagen levels in one ml of culture medium, measured by the Sircol Soluble Collagen Assay, were reported as micrograms for ml. Statistical significance across treatment groups was determined using the one-way ANOVA. A *P* value <0.05, which indicates a significant difference, is designated with an asterisk.

**Figure 8:**
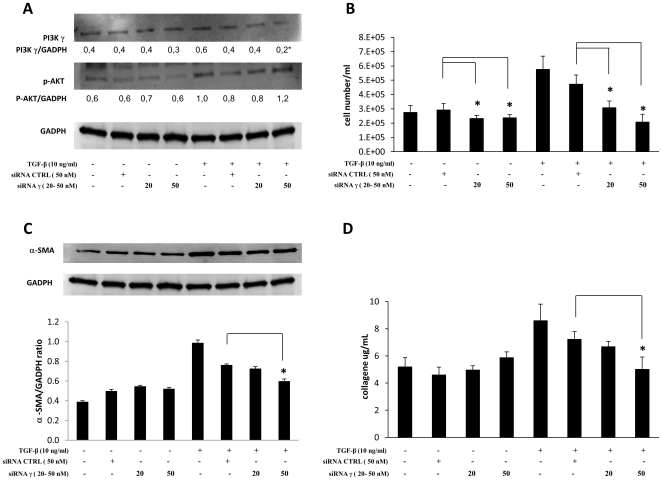
Effects of specific PI3K p110γ gene suppression by siRNA. *Ex vivo* human lung fibroblasts (from three different donors) were kept resting or transfected with small interfering RNAs (siRNAs) specifically targeting p110γ (at the concentration of 20 and 50 nM) or with negative control (50 nM), for 24 hrs in serum-free RPMI medium, afterward they were kept resting or stimulated with TGF-β (10 ng/ml) for 24 hrs in 2% FBS medium. A) Representative western blot analysis of p110α, phosphorylated (Ser^473^) Akt and GAPDH expression. with means of p110γ-/GAPDH and pAKT/α-GAPDH ratios calculated in three separate experiments B) Results of cell counting by optical microscopy. Medians and standard deviations of at least three separate counts in triplicate wells for three separate experiments are reported. C) Representative Western blot analysis of α-SMA expression and pertinent graph. Means of α-SMA/GAPDH ratios calculated in three separate experiments are indicated. D) Total soluble collagen levels in one ml of culture medium, measured by the Sircol Soluble Collagen Assay, were reported as micrograms for ml. Statistical significance across treatment groups was determined using the one-way ANOVA. A *P* value <0.05, which indicates a significant difference, is designated with an asterisk.

Figures 7 and 8 show the data of one representative experiment out of three separate transfections (in three different cell lines) with siRNA, for p110α or p110γ respectively, which obtained comparable results. As indicated by western blot analysis shown in panels A, transfections with siRNAs in unstimulated cells produced slight variations of PI3K p110 isoforms' protein levels. This finding indicates that PI3K p110 isoforms α and γ are very stable proteins (with low turnover) whose levels were only slightly affected by siRNA transfections. However, these variations caused a small but significant effect on cell proliferation (panels B). Moreover, selective gene knocking produced a 29–67% specific inhibition of TGF-β-induced increase in p110α or p110γ expression (including some newly synthesized proteins) as well as an equivalent significant suppression of TGF-β-induced increase in cell proliferation, α-SMA and collagen expression (panels C and D). On the contrary, AKT-Ser^473^ phophorylation induced by TGF-β was only marginally affected (panels A, bottom). Furthermore, it is noteworthy that, on one hand, the negative control inhibited the increase in gene expression of both PI3K p110 isoforms as well as in cell proliferation and fibrosis markers induced by TGF-β stimulation (the most plausible reason for this is that an interaction between siRNAs/siPort and TGF-β, and/or its receptors, occurred and lowered the effects of the latter); on the other hand, specific siRNAs produced significantly higher inhibitory effects compared to the negative control's.

## Discussion

The molecular pathogenetic mechanisms of IPF remain unclear and few if any efficacious therapies are available for this fatal disease. Therefore, large gaps in knowledge remain and novel antifibrotic drugs are urgently needed for its treatment. In a recent paper, by inhibiting Akt activation in human lung fibroblasts we provided evidence that phosphorylation of this serine/threonine kinase is involved in both fibroblasts proliferation and differentiation into myofibroblasts which play an essential role in fibrotic disease [12]. Moreover, it has been shown that fibroblasts isolated from IPF patients display pathological activation of Akt [17]. In this study we investigated the role of the upstream PI3Ks and we demonstrate that the TGF-β-induced proliferation of *ex-vivo* human lung fibroblasts as well as their differentiation into myofibroblasts depends on class I PI3Ks being activated. In fact, cell treatment with LY294002 (the pan-inhibitor of class I PI3Ks) was able to completely abrogate the TGF-β-induced proliferative effect as well as α-SMA expression and collagen production. Furthermore, transfections with selective siRNAs for PI3K p110α and p110γ produced similar results. This finding agrees with previously reported observations on murine cell lines [6] and with a recent study demonstrating that PI3K/Akt plays an important role in the fibrogenesis of human lung fibroblasts induced by bleomycin by up-regulating cell growth and collagen expression [18]. Moreover, we show that in addition to ubiquitously expressed p110α and β, human lung fibroblasts also express p110δ and γ thus suggesting that their expression is not restrained within the haematopoietic system, in accordance with previously published data. In particular, several observations have demonstrated that p110γ is functionally expressed in mouse cardiofibroblasts (reviewed in [19]) as well as p110δ being recently shown in human peripheral lung tissue [20]. Furthermore, by using specific pharmacological inhibitors we show a major role of PI3K p110γ and α in sustaining the TGF-β–induced increase in proliferation, yet in a context of the functional redundancy of all class I isoforms. Actually, all selective inhibitors were individually able (in varying extents) to inhibit the proliferative effect but none alone emulated the complete suppression of the pan-inhibitor LY294002. This finding supports previously reported data on mouse embryonic fibroblasts' (MEFs) model [21] demonstrating the redundancy of PI3K isoforms (at least for class IA PI3K, considering that MEFs are not believed to express class IB p110γ [22]) in sustaining cell survival and proliferation hence suggesting that targeting all class I PI3Ks is essential in producing the maximum inhibition of cell proliferation. Moreover, selective gene knocking by siRNAs was able to specifically inhibit about 30–65% of TGF-β-induced p110α or p110γ over-expression paralleled by a equivalent or more robust suppression of TGF-β-induced increase in cell proliferation as well as in α–SMA and collagen expression, therefore confirming a crucial role of both isoforms in sustaining this process and their mutual interplay.

Furthermore, in TGF-β–induced fibrogenic effects, we show a certain redundancy of class IA p110α and class IB p110γ. However, it is noteworthy that treatment with the specific p110γ inhibitor AS252424 at nanomolar concentrations much lower than the IC_50_ of the other isoforms significantly prevented α-SMA and collagen production induced by TGF-β whereas at concentrations ≥5 µM, probably also affecting p110γ, it completely abrogated these effects. Since a previously reported *in vivo* study showed that oral administration of AS605240 significantly prevented lung inflammation and reduced collagen deposition in rats [23], our novel finding suggests that the effects of AS605240 (another p110γ inhibitor) on bleomycin-induced pulmonary fibrosis could be attributed not only to suppressing inflammatory cell recruitment, as indicated in that paper, but also to a direct anti-fibrotic effect on lung fibroblasts. Importantly, we also show that the role of Akt downstream class I PI3Ks appears to be only partial in TGF-β-induced proliferation and very marginal in fibrogenic effects because, despite of the complete blockade of Akt phosphorylation achieved by e.g. TGX-221, we observed slight inhibition of the TGF-β-induced increase in proliferation and no reduction at all of α-SMA expression or collagen production. This finding was supported by results obtained with siRNA transfections. We showed that selective suppression of p110α/p110γ gene expression was able to significantly reduce the TGF-β-induced fibroblast proliferation and fibrotic response without substantially affecting AKT-Ser^473^ phophorylation. Further investigation should target other components of the PI3K signalling pathway.

Overall, our results suggest that class I PI3Ks might be considered significant new targets for treating idiopathic pulmonary fibrosis.

## Supporting Information

Figure S1 **Flow cytometry plots of one representative experiment as described in** **Fig. 1** **.** F = fibroblasts. Pertinent Mean Fluorescence Intensity (MFI) is indicated below and concentrations of LY294002 (LY) and TGF-β(TGF) are indicated above each plot.
(TIF)

Figure S2 **Flow cytometry plots of one representative experiment as described in** **Fig. 3** **.** F = fibroblasts. Pertinent Mean Fluorescence Intensity (MFI) is indicated below and concentrations of YM-024 (YM) and TGF-β (TGF) are indicated above each plot.
(TIF)

Figure S3 **Flow cytometry plots of one representative experiment as described in** **Fig. 4** **.** F = fibroblasts. Pertinent Mean Fluorescence Intensity (MFI) is indicated below and concentrations of TGX-221 (TGX) and TGF-β (TGF) are indicated above each plot.
(TIF)

Figure S4 **Flow cytometry plots of one representative experiment as described in** **Fig. 5** **.** F = fibroblasts. Pertinent Mean Fluorescence Intensity (MFI) is indicated below and concentrations of IC-87114 (IC) and TGF-β (TGF) are indicated above each plot.
(TIF)

Figure S5 **Flow cytometry plots of one representative experiment as described in** **Fig. 6** **.** F = fibroblasts. Pertinent Mean Fluorescence Intensity (MFI) is indicated below and concentrations of AS-2524224 (AS) and TGF-β (TGF) are indicated above each plot.
(TIF)
